# Editorial: Human Milk Composition and Health Outcomes in Children

**DOI:** 10.3389/fped.2019.00319

**Published:** 2019-07-30

**Authors:** Daniel Munblit, Valerie Verhasselt, John O. Warner

**Affiliations:** ^1^Department of Paediatrics and Paediatric Infectious Diseases, Institute of Child's Health, Sechenov First Moscow State Medical University (Sechenov University), Moscow, Russia; ^2^Department of Paediatrics, Imperial College London, London, United Kingdom; ^3^The In-VIVO Global Network, An Affiliate of the World Universities Network (WUN), New York, NY, United States; ^4^Solov'ev Research and Clinical Center for Neuropsychiatry, Moscow, Russia; ^5^School of Molecular Sciences, University of Western Australia, Perth, WA, Australia; ^6^National Institute for Health Research, Collaboration for Leadership in Applied Health Research and Care for NW London, London, United Kingdom

**Keywords:** allergy, breast milk, cytokines, fatty acids, human milk, human milk oligosaccharides, microbiota, milk fat globule

Human breast milk (HM) is the physiological nutrition during an infants' first few months of life, a crucial period for immune system development, metabolic and endocrine programming for growth, development, and lifelong health. HM is a complex and variable mixture with a multitude of constituents each contributing either singly or in combination to health outcomes (1). This Research Topic aims to present recent research findings on the complex relationships between HM composition and health outcomes in children, with state-of-the-art reviews, systematic analysis of published data, providing a complete presentation of current knowledge in the field.

Human infants are born with a physiologically relatively immature immune system which is partly compensated by immune active molecules in HM. Furthermore, many immune active molecules in HM have been linked with a variety of health outcomes, suggesting they may also influence the trajectory of immune development (2). Review from Rajani et al. focuses on variations in immune active components that have been reported to be associated with allergic risk in children, providing insight into potential mechanisms of action. Although cytokines and growth factors have been mostly investigated in relation to allergy development, some authors studied associations with growth patterns (3) but little is known of their impact on the growth of infants in the less affluent countries. Saso et al. found levels of cytokines in mature milk being weakly predictive of poor infant growth in Gambia, suggesting that milk compositional changes may be a consequence of suboptimal maternal health and nutrition.

Transforming growth factor beta (TGF-β) is one of the best-studied immune regulatory cytokines and often is investigated with regards to allergy development. A recent systematic review failed to find convincing evidence of associations between HM TGF-β and allergic outcomes (4). Most of the researchers have drawn conclusions based on the links between concentration of immunological markers in colostrum and/or mature milk and allergic diseases. Morita et al. used a more creative approach, looking at ratio (mature milk/colostrum) rather than single time-point concentration, reporting lower TGF-β1 ratio to be associated with the development of eczema. This indicates that innovative approaches to data analysis coupled with more extensive use of serial data could unveil links between the TGF-β and allergic diseases development.

To further understand how breastfeeding can influence immune development, Hsu and Nanan present a critical analysis of potential associations between breastfeeding and the development of thymus, playing a crucial role in T lymphocytes maturation and output.

Human milk oligosaccharides (HMOs) are as a group the 3rd largest component of HM. More than 200 HMOs are present in HM, and diversity and complexity of HMOs are very specific to HM. Both, short-and long-chain oligosaccharides are found, at a unique ratio. HMOs play an important role in microbiome development and immune system maturation thereby having short- and long-term impacts on infant health. In this Research Topic, two reviews address HMOs importance for infant health and development. Ayechu-Muruzabal et al. provide an overview of the HMOs diversity and impact on early life immune development, while Triantis et al. discusses potential effects of HMOs on infectious and non-communicable diseases and inflammation in general. This paper emphasizes the role of HMOs in altering immune responses through binding to immune-related receptors. There were very few attempts to systematically review available evidence on human milk composition and they are needed to guide future research and highlight gray areas in need of further investigation (5). In view of this, a systematic review of available data on HMOs and associations with immune-mediated disease and infection in childhood is very timely. Doherty et al. reported low concentrations of lacto-N-fucopentaose (LNFP)-III being associated with cow's milk allergy and that higher fucosyl-oligosaccharide levels provides some protection against infectious disease, however, the evidence is sparse and more studies are required.

Although HM has been considered sterile this concept has changed in the last decades with extensive research of HM microbiome. In a recent systematic review of published evidence, a total of 820 microbe species were identified in HM, mainly consisting of Proteobacteria and Firmicutes (6). Human milk oligosaccharides and HM microbiome are acting in synergy, influencing immune system development. Moossavi et al. provide a nice overview review of the latest evidence, mechanisms, and hypotheses for the synergistic and/or additive effects of milk microbiota and HMOs in relation to asthma development in children.

Metabolomic studies increase in numbers and provide complex analysis, with recent data showing distinct patterns associated with maternal lifestyle and the environment (7). Complex, but intriguing interactions between nutrients, microbiome, and metabolites in HM is comprehensively reviewed in the work from Bardanzellu et al. They outline targets for the future research, suggesting a promising approach, of integrative assessment of metabolomics, microbiomics, and HM cellular composition, as the way toward better understanding the potential health promoting properties of HM.

Attention to HM polyunsaturated fatty acids (PUFAs) in relation to health outcomes is based on the hypothesis that the modern diet has led to changes in ratio of n-6 to n-3 fatty acids which may result in increase in allergic diseases prevalence in children. This concept resulted in a number of studies assessing HM PUFAs profile association with atopy/allergy development. Recent systematic review of 18 papers from 15 study populations reported heterogeneity among studies with insufficient evidence to suggest that HM PUFAs influence the risk of childhood allergic diseases (8). This was primarily explained by lack of standardized methodology, such as differences in stage of lactation, variations in definition of allergic outcomes as well as lack of adjustment for potential confounders or even over-adjustment in some studies. Logan and Genuneit discuss the results of the systematic review in their commentary. In light of their subsequent original research (9) they suggest avenues for future research, such as employment of the centered log ratio transformation to overcome spurious correlation, considerations for alternative ways of grouping fatty acids and reduction of selection bias being of particular importance.

Diversity of HM constituents, such as immune active factors, oligosaccharides, and microbes contribute to complex interactions within the HM and associated with gut barrier function, the gut microbiota, and oral tolerance induction (10). It was hypothesized that modulation of infant gut immunity and microbiome may facilitate tolerance development and thus allergy prevention. The impact of breastfeeding on shaping the neonate's gut microbiota and preventative effect on allergy development is reviewed by van den Elsen et al. Emerging data suggests that Milk Fat Globule Membrane (MFGM) may also play in important role in structural and functional maturation of infant gut. Comprehensive review of one of the less well-studied HM constituents, factors influencing its characteristics and potential role in gut immunity maturation presented by Lee et al.

Future of HM research is tightly linked with multi-disciplinary collaborations, aiming to approach HM as a complex and dynamic fluid, which will have various impact on the short and long-term of infant health. We hope that this compilation of articles accumulating existing evidence in the field of HM research will be of an interest to the readers and will inspire more state-of-the-art work on the topics described.

## Author Contributions

All authors listed have made a substantial, direct and intellectual contribution to the work, and approved it for publication. The views expressed in the paper are those of the authors and not those of the NIHR or Department of Health (UK).

### Conflict of Interest Statement

The authors declare that the research was conducted in the absence of any commercial or financial relationships that could be construed as a potential conflict of interest.
